# Radiomics-based classification of medication-related osteonecrosis of the jaw using panoramic radiographs

**DOI:** 10.1007/s11282-025-00826-1

**Published:** 2025-05-05

**Authors:** Masaru Konishi, Hiromi Nishi, Hiroyuki Kawaguchi, Naoya Kakimoto

**Affiliations:** 1 Department of Oral and Maxillofacial Radiology, Hiroshima University Hospital, 1-2-3 Kasumi, Minami-ku, Hiroshima, 734-8553 Japan; 2 Department of General Dentistry, Hiroshima University Hospital, Hiroshima, Japan; 3 Department of Oral and Maxillofacial Radiology, Graduate School of Biomedical and Health Sciences, Hiroshima University, Hiroshima, Japan

**Keywords:** Medication-related osteonecrosis of the jaw, Radiomics, Machine learning, Panoramic radiography

## Abstract

**Objectives:**

Medication-related osteonecrosis of the jaw (MRONJ) caused by bone resorption inhibitors is difficult to treat and reduces the patient’s quality of life. We aimed to classify the likelihood of MRONJ development using panoramic radiographs taken prior to bone resorption inhibitor administration.

**Methods:**

We included patients who underwent panoramic radiographic evaluation at Hiroshima University Hospital prior to bone resorption inhibitor administration. Thirty-two patients with MRON of the mandible (16 men and 16 women) and 57 without MRONJ (23 men and 34 women) were selected. The mandible was segmented from the mental foramen to the anterior mandibular angle notch on panoramic radiographs before treatment. The image features within this region were extracted and quantified. Overall, 13 shape, 18 histogram-based, 75 texture-based, and 744 wavelet features were extracted. Least absolute shrinkage and selection operator regression were used to select relevant features from the extracted data. Support vector machine (SVM) and neural network of multilayer perceptron (MLP) were used as machine-learning models. The sensitivity, specificity, and area under the curve (AUC) from the receiver operating characteristic curves were used to evaluate diagnostic performances.

**Results:**

The SVM model achieved a sensitivity of 0.667, a specificity of 0.833, and an AUC of 0.903 in the test group. Meanwhile, the MLP model achieved a sensitivity of 0.833, a specificity of 0.750, and an AUC of 0.903 in the test group.

**Conclusion:**

Radiomics analysis of panoramic radiographs taken before bone resorption inhibitor administration can differentiate between patients with MRONJ and those without MRONJ.

## Introduction

Medication-related osteonecrosis of the jaw (MRONJ) is primarily caused by the use of bone resorption inhibitors. Marx initially reported its occurrence in patients with malignant tumors following the administration of high-dose bisphosphonates [1]. A total of 2,664,104 patients with osteoporosis received bone resorption inhibitors, with an MRONJ incidence of 1,603 (0.06%). By contrast, 155,206 patients with cancer received bone resorption inhibitors, with an MRONJ incidence of 2274 (1.47%) [2]. Although the overall incidence of MRONJ is low, advanced stages of the condition are refractory to conservative treatment and often necessitate surgical intervention, which can significantly compromise the post-treatment quality of life due to jawbone resection [3, 4].

The development of MRONJ is attributed to the inhibition of bone remodeling and angiogenesis caused by the administered drugs [5]. Although these medications, administered orally or intravenously, are intended to affect the systemic bone, MRONJ predominantly occurs in the jawbone. We hypothesize that this site-specific predisposition may be linked to the unique features of jawbone quality prior to the administration of bone resorption inhibitors.

In recent years, the field of radiomics has emerged as a promising approach, combining radiologic imaging features with machine-learning techniques. Radiologic images have been used to predict treatment prognosis [6], differentiate benign from malignant conditions [7, 8], and predict the side effects of radiotherapy [9]. In the field of dental and oral surgery, studies have focused on evaluating various applications of radiomics, including the diagnosis of cervical lymph nodes [10, 11], prediction of cervical lymph node metastasis in tongue cancer [12], and evaluation of the histopathological grade of oral squamous cell carcinoma using18-fluorine-fluorodeoxyglucose positron emission tomography images [13]. These studies have demonstrated the efficacy of radiomics. Building on these findings, we hypothesized that the radiomics analysis of panoramic radiographs could be used to predict the development of MRONJ. Accordingly, this study aimed to classify patients based on the likelihood of developing MRONJ through the radiomics analysis of panoramic radiographs obtained prior to the administration of bone resorption inhibitors.

## Materials and methods

This retrospective study was approved by the Hiroshima University Ethics Committee (approval number: E2023-0269) and was conducted in compliance with the 1975 Declaration of Helsinki, as amended in 1983. Informed consent was obtained through an opt-out method, in accordance with the guidelines of the local institutional ethics committee.

### Patients

**Fig. 1:**
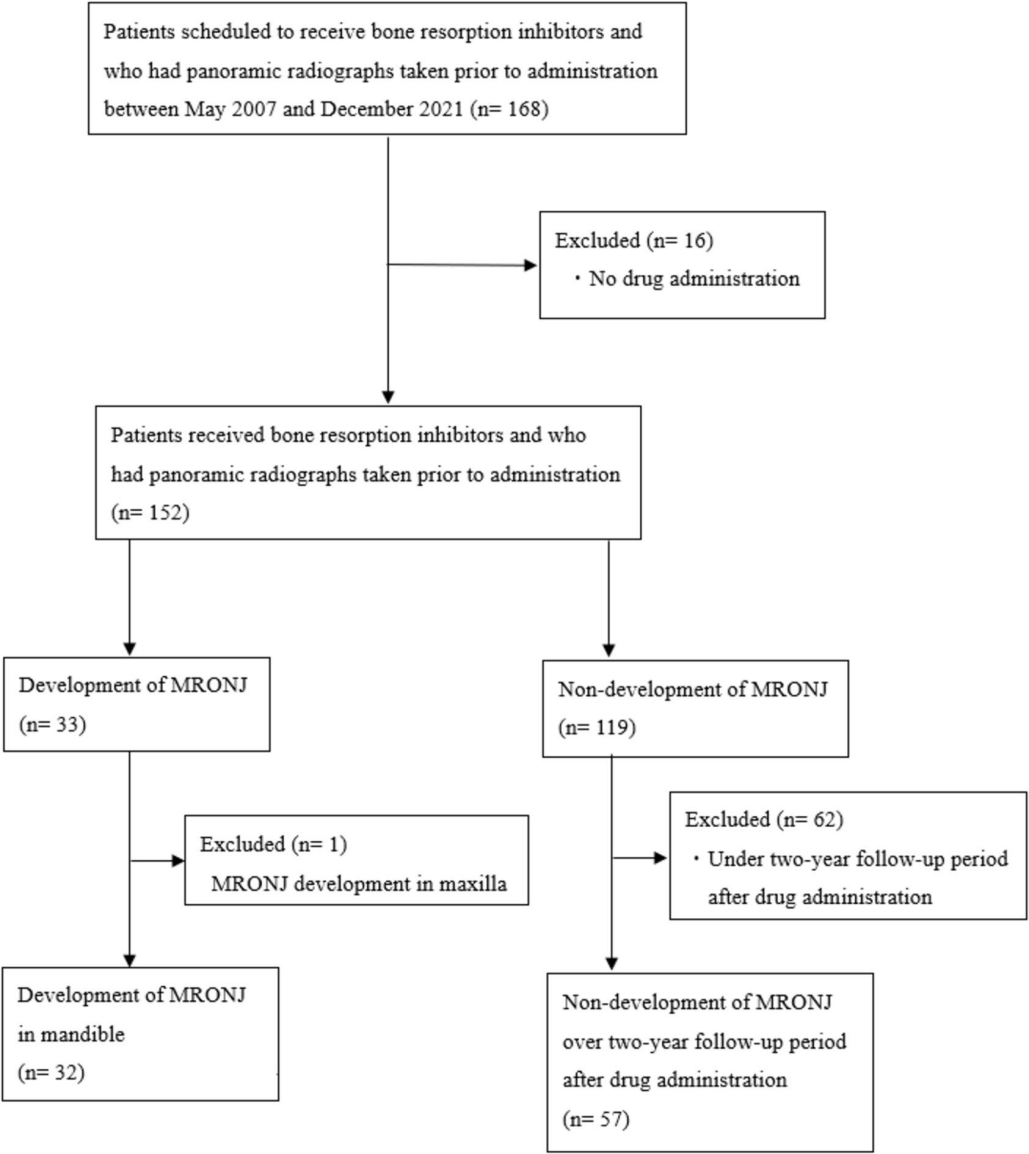
Flow diagram showing the participant selection process

The patient selection process is shown in Fig. 1. Patients scheduled to receive bone resorption inhibitors at Hiroshima University Hospital between May 2007 and December 2021 and who had panoramic radiographs taken prior to the administration were enrolled in the study. A total of 168 patients were selected, of whom 152 were confirmed to have received bone resorption inhibitors. Among the 152 patients, 33 developed MRONJ, whereas 119 did not. From the MRONJ group, 1 patient with MRONJ affecting only the maxilla was excluded, leaving 32 patients included in the final analysis as the MRONJ group. In the non-MRONJ group, patients who had been followed for less than 2 years after receiving bone resorption inhibitors were excluded. Hence, only 57 patients who had been followed for more than 2 years were included in the non-MRONJ group. The diagnosis of MRONJ was established based on the following criteria [14]: (1) a history of current or past treatment with antiresorptive therapy, either alone or in combination with immunomodulatory or angiogenesis inhibitors; (2) exposed or explorable bone through an intraoral or extraoral fistula in the maxillofacial region persisting for at least 8 weeks; and (3) no history of radiation therapy or metastasis to the jaw. Ultimately, 32 MRONJ and 57 non-MRONJ patients were included in the final analysis (Table 1). All panoramic radiographs were obtained using Hyper X or AUGE X ZIO CM (ASAHIROENTGEN IND. CO., LTD., Kyoto, Japan).

**Table 1:** Patient characteristics

Characteristics	MRONJ (*n* = 32)	Non-MRONJ (*n* = 57)	*P* value
Age, years (mean ± SD)	66.1 ± 10.4	60.8 ± 13.7	0.0802
Sex (male/female)	16/16	23/34	0.5046
Primary lesion			
Osteoporosis	4	0	-
Malignant tumor			
Breast	8	16	
Lung	6	12	
Prostate	4	6	
Kidney	4	2	
Liver	2	6	
Tongue	1	0	
Uterus	2	1	
Multiple myeloma	1	4	
Thyroid	0	2	
Biliary	0	1	
Thymus	0	2	
Bladder	0	1	
Esophagus	0	1	
Large intestine	0	1	
Giant cell tumor of bone	0	2	
Types of medication			
Bisphosphonates alone	7	27	0.0014*
Denosumab alone	20	30	
Bisphosphonates and denosumab	5	0	
Period of medication until MRONJ development, months (median, range)	20 (3–141)	–	
Period of medication until last follow-up date, months (median, range)	–	47 (24–189)	

*Indicates statistically significant difference

*MRONJ* medication-related osteonecrosis of the jaw, *SD* standard deviation

The patient data were randomly divided into the training (*n* = 53), validation (*n* = 18), and test (*n* = 18) groups for machine-learning evaluation using a statistical software (Table 2). No significant differences were found among the three groups in terms of MRONJ status, age, sex, medication type, time until MRONJ development, or follow-up period in the non-MRONJ group.

**Table 2:** Patient characteristics in the training and validation groups

Characteristics	Training group (*n* = 53)	Validation group (*n* = 18)	Test group (*n* = 18)	*P* value
MRONJ/non-MRONJ	19/34	7/11	6/12	1.0000
Age (years, mean ± SD)	63.0 ± 12.5	60.5 ± 16.3	64.1 ± 9.8	0.9765
Sex (male/female)	23/30	7/11	9/9	0.7996
Medication				
Bisphosphonate	20	7	7	0.8236
Denosumab	31	9	10	
Bisphosphonates and denosumab	2	2	1	
Period of medication until MRONJ development, months (median, range)	38 (3–137) (*n* = 13)	19 (5–141) (*n* = 7)	13.5 (5–77) (*n* = 6)	0.3787
Period of medication until last follow-up date, months (median, range)	45.5 (24–189) (*n* = 34)	31 (23–141) (*n* = 13)	57 (25–156) (*n* = 13)	0.3347

*MRONJ* medication-related osteonecrosis of the jaw, *SD* standard deviation

### Region of interest segmentation and radiomics feature extraction and selection

**Fig. 2:**
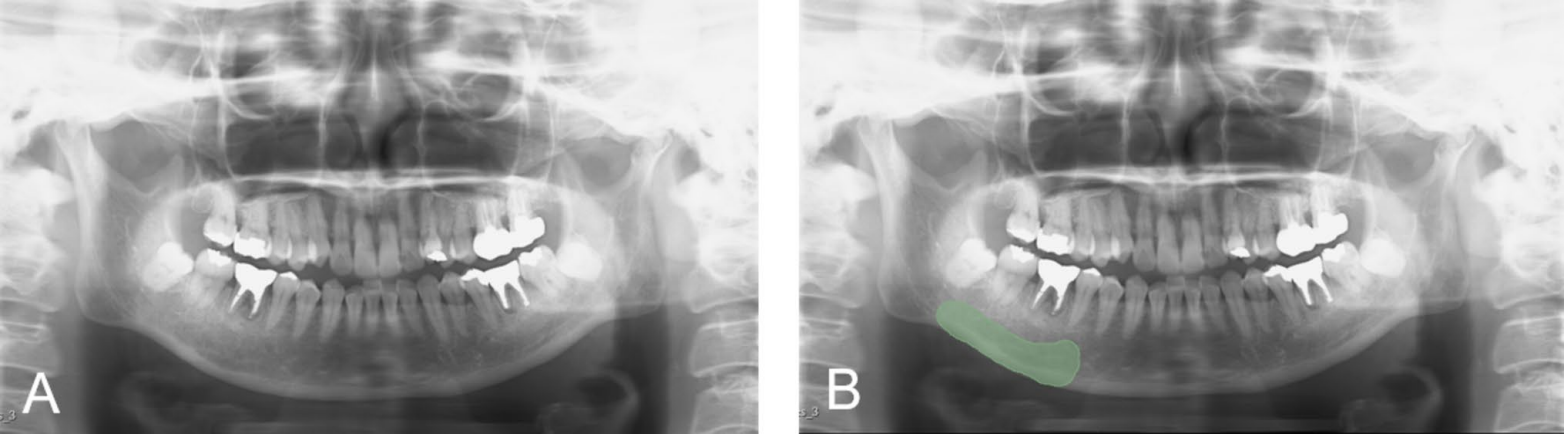
Example of segmentation using panoramic radiographs. **A** Original panoramic radiograph before treatment. **B** Segmentation of the mandible before treatment

Region of interest (ROI) segmentation of the mandible was performed on panoramic radiographs taken prior to medication administration using an open-source software, 3D slicer (https://www.slicer.org/) [15]. On panoramic radiographs, the mandible was segmented from the mental foramen to the anterior mandibular antegonial notch and from the superior border of the mandibular canal to the inferior border of the mandible (Fig. 2). The segmentation of this region was designed to avoid the influence of the teeth, the cervical spine in the anterior region, and the pharyngeal cavity and the contralateral mandibular ramus in the posterior region. The reason for including the cortical bone along the lower border of the mandible is as follows. Although MRONJ typically develops from the alveolar bone in cases of periodontitis or after tooth extraction, it can also occur from the mylohyoid muscle line or the bony prominences of the jaw [16]. MRONJ develops from either cancellous or cortical bone. Therefore, cancellous and cortical bone were included in the ROI segmentation in this study. Image features (radiomics) were extracted using the open-source software Pyradiomics package (Pyradiomics 3.0; http://pyradiomics.readthedocs.io) [17, 18] with a three-dimensional slicer. Radiomics features serve as one of the key image biomarkers [19].

Image texture analysis was used to extract and quantify image features from the region, including first-order statistical, shape-based, histogram-based, and texture-based features. These texture-based features include gray-level co-occurrence, gray-level run length, gray-level size zone, gray-level distance zone, neighboring gray-level dependence, and neighborhood gray-tone difference matrices. Higher-order statistical features were extracted using a wavelet imaging filter, incorporating low-pass and high-pass filters to reduce interference from gas [10]. The image features within the segmentation region were extracted and quantified, resulting in a total of 850 image features. These included 13 shape, 18 histogram-based, 75 texture-based, and 744 wavelet features (related to histogram and texture). To identify valid features, the least absolute shrinkage and selection operator (LASSO) was used. LASSO is the method that simultaneously performs radiomics feature selection and data fitting by building a classification model trained on the data. A leave-one-out cross-validation approach was employed for internal validation to avoid overfitting during model fitting and hyperparameter optimization [12]. In addition, an early stopping function was used to prevent overfitting.

**Fig. 3:**
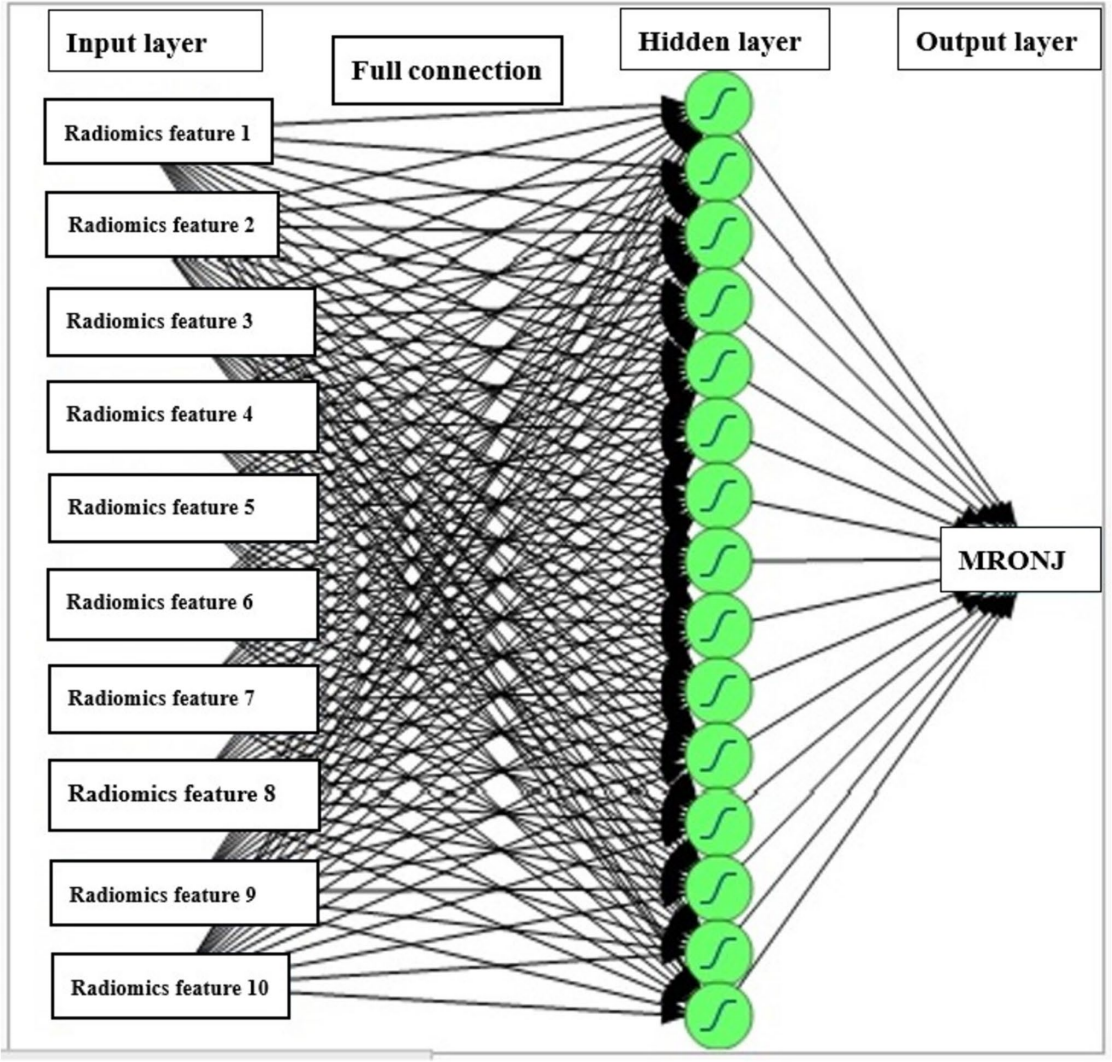
Construction of the MLP neural network. Radiomics feature 1: Maximum2DdiameterColumn. Radiomics feature 2: Wavelet-LHL_firstorder_Skewness, Radiomics feature 3: Wavelet-LHH_firstorder_Skewness, Radiomics feature 4: Wavelet-HHL_firstorder_Range, Radiomics feature 5: Wavelet-LLL_firstorder_Maximum, Radiomics feature 6: Wavelet-LHH_GLSZM_ HighGrayLevelZoneEmphasis, Radiomics feature 7: Wavelet-LHH_GLSZM_ LowGrayLevelZoneEmphasis, Radiomics feature 8: Original_GLRLM_RunLengthNonUniformity, Radiomics feature 9: Wavelet-HLH_GLRLM_ HighGrayLevelRunEmphasis, Radiomics feature 10: Wavelet-HLH_GLRLM_ LowGrayLevelRunEmphasis

Support vector machine (SVM) and neural network of multilayer perceptron (MLP) were used as machine-learning models [12]. The models were evaluated for sensitivity, specificity, accuracy, and precision. The area under the curve (AUC) was evaluated using the receiver operating characteristic (ROC) curve. The MLP model, which utilized backpropagation on the measurements to classify the likelihood of developing MRONJ, consisted of an input layer, an output layer, and a hidden layer (intermediate layer). The TanH function was employed as the activation function in this study. All neurons in the input, hidden, and output layers were connected (Fig. 3). In addition, a boosting method was implemented, in which several small models (base models) were fitted sequentially, and their results were combined to create a composite model. During boosting, a small model is fitted, and its residuals (scaled residuals) are calculated. The residuals are then refitted to the subsequent small model in an iterative process. Finally, the small models are combined to produce the final model. The model was evaluated using the validation set to determine the number of iterations for fitting the smaller models. The boosting performance was set to 6 models and a training rate of 0.1. MLP classification was conducted using JMP Pro (version 18.0; SAS Institute, Cary, NC, USA).

### Statistical analyses

Statistical analyses were performed using the Wilcoxon rank-sum test for numerical data and Chi-square or Fisher’s exact tests for nominal data. All statistical analyses were carried out using JMP Pro (version 18.0; SAS Institute, Cary, NC, USA). A *P* value of < 0.05 was considered significant.

## Results

**Fig. 4:**
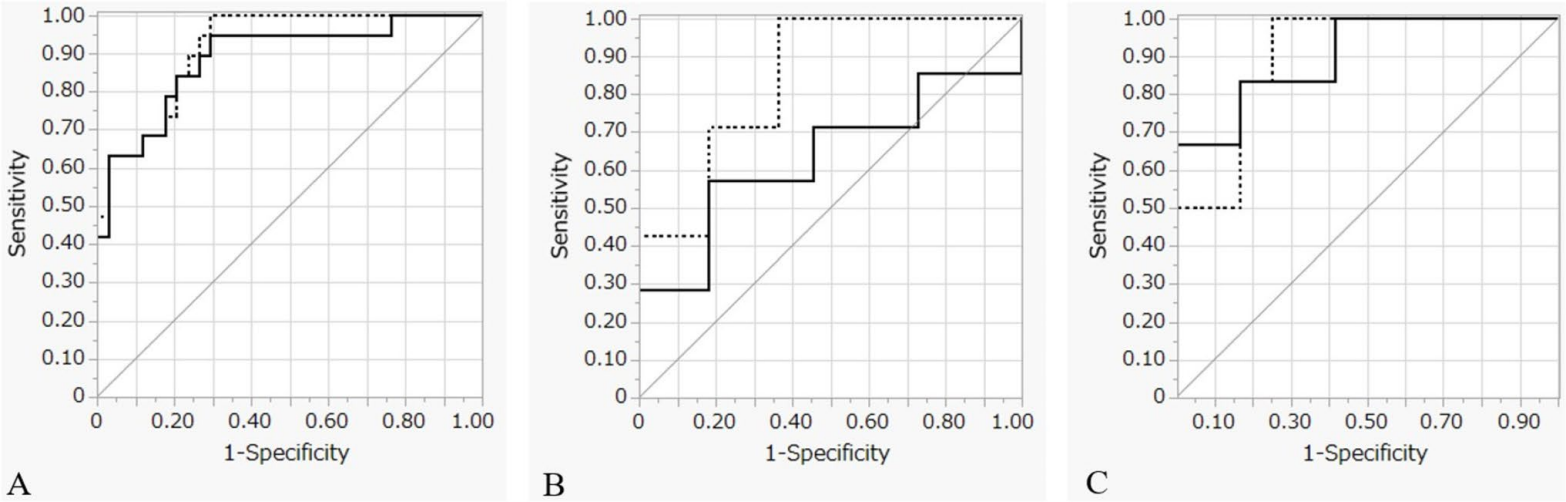
ROC curves of the machine learning models using SVM and MLP. ROC curves of the SVM and MLP models in the **A** training, **B** validation, and **C** test groups. Solid line, SVM; dotted line, MLP. ROC, receiver operating characteristic; SVM, support vector machine; MLP, neural network of multilayer perceptron

The MRONJ and non-MRONJ sides were segmented using panoramic radiographs obtained prior to treatment, and 850 image features were extracted and quantified from each site. LASSO regression analysis was performed on the extracted image features, resulting in the selection of ten key features (Table 3). The performance of the machine-learning models, including SVM and MLP, and their corresponding ROC curves, based on these ten different image features, are presented in Table 4 and Fig. 2. The sensitivity, specificity, accuracy, precision, and AUC values of the SVM and MLP models across the three groups are provided in Table 4. The sensitivity, specificity, accuracy, precision, and AUC values of the SVM model were 0.632, 0.941, 0.830, 0.727, and 0.889 in the training group; 0.286, 0.818, 0.611, 0.500, and 0.636 in the validation group; and 0.667, 0.833, 0.778, 0.556, and 0.903 in the test group, respectively. The sensitivity, specificity, accuracy, precision, and AUC values of the MLP model were 0.632, 0.882, 0.792, 0.714, and 0.916 in the training group; and 0.571, 0.818, 0.722, 0.500, and 0.844 in the validation group; and 0.833, 0.750, 0.778, 0.500, and 0.903 in the test group, respectively (Fig. 4).

**Table 3:** Radiomics features identified using LASSO regression analysis (*n* = 10)

Feature type		Radiomics features
Category	Subcategory	
Morphologic features	–	Maximum2DDiameterColumn
First-order features	Intensity histogram	Wavelet-LHL_firstorder_Skewness
First-order features	Intensity histogram	Wavelet-LHH_firstorder_Skewness
First-order features	Intensity histogram	Wavelet-HHL_firstorder_Range
First-order features	Intensity histogram	Wavelet-LLL_firstorder_Maximum
Texture-based features	Gray level size zone matrix	Wavelet-LHH_GLSZM_HighGrayLevelZoneEmphasis
Texture-based features	Gray level size zone matrix	Wavelet-LHH_GLSZM_LowGrayLevelZoneEmphasis
Texture-based features	Gray level run length matrix	Original_GLRLM_RunLengthNonUniformity
Texture-based features	Gray level run length matrix	Wavelet-HLH_GLRLM_HighGrayLevelRunEmphasis
Texture-based features	Gray level run length matrix	Wavelet-HLH_GLRLM_LowGrayLevelRunEmphasis

*LASSO* least absolute shrinkage and selection operator

**Table 4:** Evaluation of machine-learning models in the training, validation, and test groups

	Training group (*n* = 53)					Validation group (*n* = 18)					Test group (*n* = 18)				
	Sens	Spec	Acc	Prec	AUC	Sens	Spec	Acc	Prec	AUC	Sens	Spec	Acc	Prec	AUC
SVM	0.632	0.941	0.830	0.727	0.889	0.286	0.818	0.611	0.500	0.636	0.667	0.833	0.778	0.556	0.903
MLP	0.632	0.882	0.792	0.714	0.916	0.571	0.818	0.722	0.500	0.844	0.833	0.750	0.778	0.500	0.903

*Acc.* Accuracy, *AUC* area under the curve, *SVM* support vector machine, *MLP* neural network of multilayer perceptron, *Prec.* Precision, *Sens.* Sensitivity, *Spec.* specificity

## Discussion

This study performed a radiomics analysis using panoramic radiographs obtained prior to the administration of bone resorption inhibitors to differentiate between MRONJ development and non-MRONJ development. Ten image features were identified as being related to MRONJ development. Among these, five were texture related, four were histogram related, and one was morphology related.

The SVM and MLP machine learnings performed using these ten image features achieved an AUC value of 0.903 in the test groups, demonstrating a high probability of differentiating between MRONJ development and non-development. This result suggests that there is a difference in bone condition between patients who will develop MRONJ and those who will not develop MRONJ before bone resorption inhibitor treatment.

A gray level run length matrix (GLRLM) and gray level size zone matrix (GLSZM) were used for machine learning to differentiate MRONJ development. Regarding studies on MRONJ, there were no reports of analyses of radiomics features using panoramic radiographs; however, one study using multidetector computed tomography (CT) reported that GLRLM and histogram features differed significantly in mandibles of patients with stage 0 MRONJ and non-MRONJ [20]. Regarding MRONJ texture analysis studies, one study used magnetic resonance imaging (MRI) to perform texture analysis of the mandible; however, owing to the use of MRI, the findings this study cannot be considered identical to the results of our study on panoramic radiographs [21]. Furthermore, this previous study investigated the histopathological relationship between radiomics features and the jawbone and identified GLRLM as a valuable imaging feature for differentiating cases of acute osteomyelitis of the jaw [21]. Therefore, GLRLM can be a potential marker that can distinguish between the bone status of the patients with MRONJ and non-MRONJ. Although not investigated by studies on MRONJ, GLRLM is useful for differentiating odontogenic cysts from odontogenic tumors [22]. Meanwhile, GLSZM has been linked to the differentiation between these two conditions [22]. These features indicate substantial grayscale variations within the segmentation area, a large difference in adjacent pixel values, and a wide range of grayscale intensities. In the current study, GLSZM contributed to the classification of patients with MRONJ, suggesting that there may be differences in the jawbone condition between patients with and without MRONJ, even before the administration of bone resorption inhibitors.

Diagnostic imaging studies of MRONJ have largely focused on early diagnosis [21, 23–26] and distinguishing MRONJ from other forms of osteomyelitis, such as infectious and radiologic types [27]. Thus, most studies have focused on the characteristics during early diagnosis using CT [28], MRI [29], and scintigraphy [30] images obtained between the administration of bone resorption inhibitors and MRONJ onset. By contrast, our study focused on panoramic radiographs obtained before the administration of bone resorption inhibitors to investigate the differences between patients who developed MRONJ and those who did not. The findings suggest that radiomics features derived from pre-treatment panoramic radiographs could serve as potential biomarkers for predicting MRONJ development. Currently, patients are often referred to dentists for oral care and dental checkups prior to the administration of bone resorption inhibitors, such as bisphosphonates and denosumab, making panoramic radiographs a common diagnostic tool. If MRONJ onset can be predicted with higher accuracy using panoramic radiographs taken during pre-treatment, this approach could be widely adopted due to the accessibility and cost-effectiveness of panoramic radiographs compared with multidetector CT and MRI. Predicting MRONJ risk from panoramic radiographs could help guide decisions on the type of medication prescribed and the intensity of dental treatment and oral care.

This study has some limitations. First, the sample size was small, which limited the ability to perform robust statistical analyses. Owing to the retrospective nature of this study, panoramic radiographs were not consistently obtained for all patients prior to the administration of bone resorption inhibitors. Second, the small sample size may have contributed to overfitting during machine-learning analyses. In addition, patient characteristics, including medication type, were not matched between the MRONJ and non-MRONJ groups owing to the sample size. Third, segmentation should ideally be performed by setting ROIs on panoramic radiographs before the administration of bone resorption inhibitors in areas that coincide with the site of MRONJ development (osteolytic or osteosclerotic changes). However, in cases where sclerotic changes and osteolytic changes are mixed in a complex manner and cannot be clearly distinguished from sclerotic or osteolytic changes. Furthermore, there were cases where the patient was diagnosed with MRONJ, but the panoramic images showed almost no obvious changes. There were also cases where panoramic radiographs were not performed after MRONJ onset, making it difficult to identify the extent of the MRONJ on the panoramic images. In these cases, the location of MRONJ could not be clearly segmented as an ROI. Therefore, in this study, it was difficult to segment the included cases by setting ROIs separately for areas of osteosclerotic change and for areas of osteolytic change. Finally, the segmentation performed on the panoramic radiographs focused on the area below the mandibular canal, excluding the roots of the teeth and avoiding overlaps with the cervical vertebrae, hyoid bone, contralateral mandibular ramus, and pharyngeal space. Segmentation in this study was performed from the superior border of the mandibular canal to the inferior border of the mandible. Consequently, the extracted image features included information on trabecular bone and inferior border cortical bone of the mandible but excluded information on the alveolar bone region. This segmentation approach did not enable the examination of the specific effects of MRONJ development in each detailed region, such as trabecular, cortical, and alveolar bone. Further studies will be conducted to further subdivide the ROI and evaluate the effect of each site on the development of MRONJ.

In conclusion, radiomic analysis and machine-learning models using panoramic radiographs demonstrated high accuracy in differentiating MRONJ from non-MRONJ. This study addresses a gap in the availability of preemptive diagnostic methods using accessible radiographic imaging and advanced analytical tools. The study is novel in its utilization of radiomics features extracted from panoramic radiographs combined with machine-learning models to predict MRONJ risk. This approach integrates advanced image analysis and artificial intelligence, offering a new perspective in the field of dental imaging and risk stratification. The study findings contribute to the development of non-invasive predictive tools for MRONJ risk; the identification of patients at risk of MRONJ before initiating bone resorption inhibitors could enable early interventions or alternative treatment plans. However, further investigations involving a larger sample size are needed to validate the findings of this study. 

## Data Availability

The datasets generated and/or analyzed during the current study are not publicly available due to the ethical restrictions but are available from the corresponding author on reasonable request.

## Declarations

### Trial registration number and date of registration

This retrospective study was approved by the Hiroshima University Ethics Committee (approval number: E2023-0269, date of registration: March 21st, 2024).
